# Misplaced Intuitions in Interventions to Reduce Attractiveness-Based Discrimination

**DOI:** 10.1177/01461672221074748

**Published:** 2022-02-18

**Authors:** Jordan R. Axt, Juanyu Yang, Harshadaa Deshpande

**Affiliations:** 1 McGill University, Montreal, Quebec, Canada; 2 Project Implicit, Seattle, WA, USA

**Keywords:** discrimination, bias, judgment, decision-making, intuition, physical attractiveness

## Abstract

Individuals and organizations are increasing efforts to address discrimination. Nonexperts may lack awareness of, or are resistant to, scientifically informed strategies for reducing discrimination, instead relying on intuition. Five studies investigated the accuracy of nonexperts’ intuitions about reducing discrimination concerning physical attractiveness. In Studies 1a to 1c (*N* = 902), participants predicted the effectiveness of six interventions to reduce attractiveness-based favoritism on a judgment task. Studies 2a and 2b (*N* = 6,292) investigated the effectiveness of these interventions. Although two interventions reduced discrimination, intuitions were poorly aligned with actual results; fewer than 1% of participants identified the combination of interventions that did, versus did not, impact judgment, and responses were more likely to be below than above chance when predicting each intervention’s effectiveness. Although follow-up work should investigate the accuracy of intuition in other forms of discrimination, these results further stress the need for greater development and adoption of evidence-based strategies for combating discrimination.

Compared with stereotypes, which are cognitive associations between groups and attributes, and prejudices, which are affective attitudes toward groups, discrimination is the behavioral output that generates and sustains disparities in real-world outcomes (Fiske, 1998). Field and laboratory research show that discrimination is pervasive in the general population and among professionals (e.g., Crosby et al., 1980; Dovidio & Gaertner, 2000; Moss-Racusin et al., 2012).

One prominent form of discrimination is based in physical attractiveness. Physical attractiveness is unlike other forms of discrimination, such as those based in race or gender, as attractiveness does not have a strong ingroup versus outgroup component; in fact, prior work suggests that people have minimal insight into how others rate them in terms of attractiveness (Kenealy et al., 1991). At the same time, people with higher or lower levels of physical attractiveness share a characteristic that has repeatedly been shown to impact real-world treatment (Hosoda et al., 2003). For instance, physically attractive people are more likely to receive job offers and promotions (Maestripieri et al., 2017), and are less likely to be fired (Commisso & Finkelstein, 2012). More attractive people also attend more coveted academic programs and achieve greater scholarly impact (Hale et al., 2021). Higher levels of physical attractiveness have even been associated with more moderate criminal sentences (Downs & Lyons, 1991). Favoritism toward more physically attractive people persists even among more experienced evaluators (Marlowe et al., 1996). In all, the benefits of being physically attractive are present in many highly consequential areas of life.

Increasingly, individuals and organizations are tasked with addressing and reducing biases in judgment and evaluation that are related to physical attractiveness. For example, prominent businesses such as LinkedIn (Recruitics, 2021) and Twitter (Shankland, 2021) have sought changes in their platforms to reduce attractiveness-based discrimination. Ideally, in these cases, organizations could look to academic research for what changes or interventions to implement, but this approach can be significantly complicated by studies relying on different populations, procedures, and outcomes. More importantly, whereas past research has identified several bias-reducing interventions in a variety of domains that have been successful in lab contexts, the current research literature lacks evidence for a suite of flexible intervention strategies that can be applied to reducing multiple forms of discrimination in both the lab and the field (e.g., Chang et al., 2019; Dobbin et al., 2007), although exceptions to this claim include the practice of “blinding” applications by completely removing irrelevant social information (Goldin & Rouse, 2000).

Without proper scientific guidance, decision-makers may have to rely on their intuitions about how to debias judgments that are possibly influenced by physical attractiveness. However, using intuition (i.e., instinctive feelings not fully informed by empirical evidence) may prioritize solutions that feel impactful, but could be unlikely to successfully address attractiveness-based discrimination, given prior work on people’s limited ability to accurately predict the causes of various psychological processes (e.g., Nisbett & Wilson, 1977). As a result, many strategies to reduce biased behavior may fall short of expectations, disappointing those who oversaw their implementation and allowing discrimination to persist.

In fact, individuals may have poor awareness for how to reduce discrimination, as suggested by prior work highlighting a general lack of accurate insight or misplaced confidence in irrelevant inputs for decision-making. For example, 47% of human resources (HR) professionals in a recent sample agreed with the notion that handwriting analyses are useful in predicting job success (Fisher et al., 2020) although prior studies have found no consistent association between handwriting and performance (Schmidt & Hunter, 1998; Simner & Goffin, 2003). Moreover, HR professionals show a reliance on intuition in strategies for determining employee evaluation, despite the availability of clear strategies for improving judgment. For instance, whereas structured interviews have been shown to reduce biases in hiring and promotion (McDaniel et al., 1994), a survey of hiring managers still found a strong preference for unstructured over structured interviews (*d* = 1.37; van der Zee et al., 2002).

Although such work reveals areas in which individuals, even those with supposed expertise in interpersonal evaluation, suffer from inaccurate intuitions about how to best improve judgment, no studies have extended this issue to addressing discrimination specifically. Furthermore, although many studies suggest high levels of inaccuracy in judgments relying on intuition, the personal importance that many individuals attach to appearing nonprejudiced (e.g., Plant & Devine, 1998) may lead to a heightened motivation for accuracy in this context, creating greater insight in the domain of discrimination relative to those covered in prior work.

To explore this issue, we asked participants to view six bias-reducing interventions based on prominent strategies in the academic literature, and to predict how those interventions would fare when improving performance on a task known to reliably create discrimination based on physical attractiveness. Next, we tested the actual effectiveness of these interventions through a large-scale comparative test using the same outcome measure. Given the consequences of such discrimination, data concerning whether nonexperts have any insight into the effectiveness of different bias-reduction interventions carries practical and theoretical implications.

From a practical perspective, results finding a lack of insight into what interventions reduce attractiveness-based discrimination will only strengthen calls for greater development and testing of scientifically backed interventions. Theoretically, such results can add to existing models of biased judgment. For example, the Wilson and Brekke (1994) model of “mental contamination” posits that bias can be avoided when decision-makers have the ability to properly adjust their responses. If participants show high levels of accuracy in predicting interventions that do or do not reduce discrimination, it would suggest introspective clarity in understanding what changes or strategies allow people to effectively adjust their behavior when confronted with the possibility of bias. Such results would then suggest that bias primarily arises from an inability to implement these changes (e.g., due to a lack of awareness, fatigue, or distraction).

Conversely, low accuracy in identifying effective interventions would illustrate that, just as people often lack insight into information that biases their judgment, so too do they lack insight into the strategies that remove the influence of such information. In this case, biased behavior would arise not only among people who lack awareness into how their behavior is being impacted, but also among many of those who successfully notice that their decisions may be biased but then employ ineffective strategies in reducing this bias.

## Measuring Discrimination in Social Judgment

To test intervention effectiveness, participants completed a measure called the Judgment Bias Task (JBT; Axt et al., 2018). In a JBT, people evaluate different profiles with relevant or irrelevant criteria for a particular outcome. In one version of the JBT, participants are on the selection committee for a hypothetical academic honor society and are given a series of applicant profiles to accept or reject. Profiles contain relevant information, such as grade point average (GPA) and interview score, and irrelevant social information, such as applicant political affiliation, race, or a photo signaling physical attractiveness.

Bias is often measured using a within-subjects approach by adopting a signal detection theory analysis in comparing the criterion values for accepting applicants from the two social groups represented in the task (e.g., more vs. less physically attractive people); a lower criterion value for an acceptance decision toward one group over another group indicates that, despite the two groups having the same overall level of qualifications, applicants from one group were held to a more lenient standard for admission. Furthermore, when coupled with self-report measures, those participants who after the task reported not wanting to use social information in their judgments or reported *having* not used social information in their judgments still showed biases in the task on average, indicating that the JBT can detect discrimination that exists outside of conscious awareness or control (Axt et al., 2018).

This work focused specifically on biases related to physical attractiveness as it was a form of bias that is produced consistently on the JBT (Axt et al., 2019; Axt & Lai, 2019). Given the clear evidence of appearance-based discrimination across many consequential contexts, physical attractiveness is a sensible domain of study for investigating the accuracy of intuitions about bias reduction. Here, we use a primarily between-subjects approach by testing the effectiveness of various intervention strategies for reducing the degree of discrimination exhibited on the JBT.

## Candidate Interventions

We identified six interventions that were previously found to either reduce bias in judgment or improve reasoning, although not all have been applied to social judgment or physical attractiveness specifically.

### Accountability

Accountability refers to the expectation that one may need to justify their beliefs, attitudes, or actions to others (Lerner & Tetlock, 1999). There are different methods for imposing accountability, including through external evaluation, reason-giving, or the mere presence of another person. Multiple studies have found that imposing accountability can significantly reduce judgment biases (e.g., Palmer & Feldman, 2005; Paolini et al., 2009; Vieider, 2009), but only if accountability was heightened before completing the relevant task and if biases were attributed to the failure to use all relevant cues and attend to one’s decision processes (e.g., Kennedy, 1993). In particular, accountability may create impression management concerns that trigger preemptive self-criticism that partly shields people from mindlessly applying simple heuristics (Lerner & Tetlock, 1999).

### Educating About Confirmation Bias

Confirmation bias is the tendency to seek out information consistent with existing beliefs. In the context of the JBT, confirmation bias may lead participants to focus on the relative strengths of more physically attractive candidates while ignoring weaknesses. Understanding and suppressing confirmation bias may then be helpful in reducing bias. A recent study (Sellier et al., 2019) found that an effortful intervention centered around educating participants about confirmation bias reduced reasoning errors on a complex business case although other instances of alerting people to biasing information have not always been successful (e.g., Jaeger et al., 2020). Still, learning about confirmation bias before completing the JBT could help reduce discrimination on the task.

### Delay

Rational, unbiased decision-making takes time, and allocating less time than is needed for judgment causes “time stress,” which can increase reliance on heuristics and lessen the amount of information considered (Ariely & Zakay, 2001; Chandler & Pronin, 2012). In prior work, greater time pressure when completing the First-Person Shooter Task (FPST), a decision-making task where participants try to quickly identify guns or harmless objects in the hands of Black or White targets, caused participants to exhibit stronger race-based discrimination (Axt & Lai, 2019). A follow-up study found that the reverse strategy—requiring a 4,500 ms delay in responding—increased accuracy and reduced discrimination on the JBT (Axt & Lai, 2019), with similar results also being found in the FPST (Correll et al., 2015).

### Implementation Intentions

Implementation intentions are a self-regulatory strategy that uses “if-then” statements to achieve certain goals, and rehearsing such statements has been shown to improve decision-making. In one study (Mendoza et al., 2010), participants completed an FPST, but beforehand half rehearsed a distraction-inhibiting implementation intention (“If I see a person, then I will ignore his race!”). Participants who rehearsed these implementation intentions showed less racial bias on the task, an outcome that is possibly explained by facilitating the automatic initiation of goal-directed behaviors without conscious reflection (Amodio et al., 2007). Implementation intentions have also been shown to be effective, at least in the short term, for reducing other instances of more automatic social biases (e.g., Lai et al., 2014; Webb et al., 2012).

### Objectivity

Committing to unbiased behavior and listing desired judgment criteria in advance may create a need for greater consistency between one’s values and actions. Some evidence for the effectiveness of this approach can be found in Uhlmann and Cohen (2005). Here, participants evaluated a candidate for police chief who was either male or female, and the applicants’ strengths were either described as being streetwise or well educated. In control conditions, results found that the hiring criteria emphasized in judgment (either being streetwise or well educated) were consistent with whatever trait was paired with the male applicant, meaning participants favored the male applicant regardless of actual qualifications. However, when participants were asked to rate the importance of each criterion *prior* to learning the applicants’ gender, there was no significant difference in the evaluation of male and female applicants. A similar approach that guides participants to think more deeply about applicant qualifications before judgments are made has also been successful, specifically in reducing biases rooted in physical attractiveness (Cann et al., 1981). Highlighting and committing to criteria before decisions are made may then be helpful when judgments are ambiguous.

### Reward

A final strategy is a reward for accurate, unbiased behavior. Although rewards are not always associated with improved performance (Read, 2005), prior studies have shown that the opportunity to receive a performance-contingent financial incentive may increase motivation or ability to regulate bias and improve overall decision-making (e.g., Enke et al., 2020; Lawson et al., 2020; Stone & Ziebart, 1995; Tosi et al., 1997).

## The Present Work

This work investigated the accuracy of intuitions about reducing socially biased judgment. In Studies 1a to 1c, we presented participants with a summary of the JBT and the biases it is known to produce. Participants were then presented with the candidate interventions and judged whether each would reduce discrimination based on physical appearance. In Studies 2a and 2b, participants were randomly assigned to a control condition or one of the six interventions, allowing us to evaluate the degree to which actual intervention effectiveness aligned with participant intuitions. Results can reveal whether nonexperts, who are increasingly responsible for addressing bias in their lives and workplaces, have any insight into what strategies work best for reducing discrimination.

## Studies 1a to 1c

### Method

#### Participants

In Study 1a, 338 participants recruited through Cloud Research completed the study on Mechanical Turk (42.5% female, 77.5% White, *M*_age_ = 37.6, *SD* = 11.6) and passed two attention check items. This sample provided 80% power to detect an effect size as small as *r* = .15 (see https://osf.io/23q8e/ for Study 1a’s preregistration). In Study 1b, 349 participants from Cloud Research (43.3% female, 80.8% White, *M*_age_ = 37.2, *SD* = 11.1) and in Study 1c, 215 participants from Prolific (66.4% female, 45.8% White, *M*_age_ = 25.3, *SD* = 6.6) completed the study and passed the same two attention check items. See https://osf.io/uxrcs/ for the online supplemental material as well as data, materials, and analysis syntax for all studies. All measures, manipulations, and exclusions are disclosed.

#### Procedure

In each study, participants first completed a five-item demographics questionnaire reporting age, gender, race, ethnicity, and political orientation. Afterward, participants read a 150-word summary of the JBT, which was described as a task that required making admissions decisions for a hypothetical academic honor society. Applicants were either objectively more or less qualified and, for each level of qualification, applicants were evenly split between more and less physically attractive people. Next, participants viewed three sample JBT applications for 5 s each. Participants were then told about typical JBT performance, which found favoritism toward physically attractive applicants, regardless of qualification (see online supplemental material for full wording of all instructions). Specifically, participants were told that, on average, a physically attractive applicant was about 9% more likely to be accepted into the honor society despite having the same level of qualifications as a less physically attractive applicant (data calculated from Axt et al., 2018).

Next, participants were told that researchers were about to test six interventions to reduce this favoritism, and it would be their job to review each intervention and predict whether it would reduce the bias favoring more physically attractive people. Participants were told that interventions would be administered immediately before people started evaluating applicants.

Afterward, in a randomized order, participants viewed the materials for each of the six interventions and were required to spend a minimum of 20 s on each page. These interventions included (a) raising accountability (being warned that researchers would review performance to probe for any errors), (b) committing to objectivity (writing down what criteria would be used in evaluation and why those criteria are important), (c) creating implementation intentions to ignore looking at an applicant’s face, (d) reviewing a short lesson about confirmation bias, (e) requiring a 4-s delay in responses, or (f) rewarding performance by making a US$5 donation to a charity of a participant’s choice if the participant was in the top 10% of accuracy on the JBT (see online supplemental material for wording of all interventions).

Participants then completed several items dealing with their perceptions about intervention effectiveness. The first item involved moving the interventions (presented with a short reminder text) into a rank order of overall effectiveness. Next, participants indicated which of the six interventions would reduce discrimination at all. An oversight in the programming of Study 1a required participants to select at least one intervention to be effective, so an option was added in Studies 1b and1c, which allowed participants to indicate whether they believed none of the interventions would be effective. Finally, in Studies 1a and 1b, participants completed the rank order item but for how they felt *other* participants would rank the interventions.

### Results

Table 1 presents the average rank of each intervention and the percentage of participants who believed that each intervention would be effective. In general, results showed considerable variability across participants; for example, the intervention most frequently predicted to be most effective was committing to objectivity, but only 24% of participants held that view in Study 1a, 27.5% in Study 1b, and 31.7% in Study 1c. In addition, there were 58 unique combinations of interventions that were believed to be effective in Study 1a, 54 in Study 1b, and 51 in Study 1c.

**Table 1.** Average Rankings and Percentage Selected as Effective for Each Intervention.

Intervention	Study 1a		Study 1b		Study 1c	
	Average rank	% effective	Average rank	% effective	Average rank	% effective
Accountability	3.34^c^	47.0	3.42^c^	47.9	3.40^c^	48.6
Objectivity	2.84^a^	63.3	2.66^a^	60.7	2.49^a^	79.9
Implementation intentions	3.04^a,b^	63.3	3.13^b^	52.1	3.18^b,c^	59.8
Confirmation bias	3.20^b,c^	58.6	3.23^b,c^	49.9	2.98^b^	60.7
Delay	4.24^d^	28.1	4.24^d^	26.1	4.13^d^	36.0
Reward	4.33^d^	24.9	4.33^d^	30.7	4.81^e^	22.9

*Note*. Interventions sharing a superscript letter did not reliably differ from each other (see online supplemental material for results of each test).

Still, a series of Wilcoxon signed rank tests (used to compare average rankings) revealed consistent patterns between the three samples. In all studies, committing to objectivity was perceived as the most effective intervention (although this did not reliably differ from implementation intentions in Study 1a). Implementation intentions and confirmation bias were either the second or third most effective interventions, and these two did not reliably differ from each other. In Studies 1a and 1b, rankings of confirmation bias and accountability did not reliably differ from each other although, in Study 1c, accountability was ranked reliably lower. For all studies, delay and accuracy rewards were the lowest ranked interventions and only in Study 1c was accuracy reward ranked reliably lower than delay. See the online supplemental material for results of each test comparing average rankings between interventions.

### Discussion

Overall, participants were confident that several of the intervention strategies would be effective at reducing attractiveness-based discrimination. For example, 61.5% of participants in Study 1a, 55% of participants in Study 1b, and 70.6% of participants in Study 1c thought that three or more interventions would be effective. In all, the three studies showed consistency in what intervention was deemed most effective (committing to objectivity), what interventions were ranked as more moderately effective (reading about confirmation bias or increasing accountability), and what interventions were ranked as the least effective (rewarding accuracy through charitable donations or requiring a delay in responses).

In Studies 2a and 2b, we directly tested how well these predictions aligned with actual effectiveness by randomly assigning participants to receive one of the six interventions. In particular, we adapted a signal detection theory (Green & Swets, 1966) approach and considered an intervention to be successful if, relative to a control condition, the intervention either reduced criterion biases favoring more physically attractive applicants (i.e., lessening the degree to which errors favored more attractive applicants) or increased overall task sensitivity (i.e., reducing the total number of errors made in evaluation) as both of these outcomes have been shown to determine the magnitude of discrimination on the task (Axt & Lai, 2019).

## Studies 2a and 2b

### Method

#### Participants

In Study 2a, 4,011 volunteer participants from the Project Implicit research pool (64.5% female, 68.7% White, *M*_age_ = 35.25, *SD* = 15.40) completed at least the JBT. Participants were excluded from analysis if they accepted less than 20% or more than 80% of applicants and if they accepted or rejected every more or less physically attractive applicant (Axt et al., 2018) as such behavior suggests participants may have disregarded JBT instructions. This left 3,723 eligible participants, which provided greater than 90% power for detecting a between-subjects effect as small as Cohen’s *d* = .225 (see https://osf.io/fq4vb/ for Study2a’s preregistration).

In Study 2b, 2,714 participants were recruited from Prolific Academic and were paid GBP £1 for the completion of the study (39.2% female, 83.8% White, *M*_age_ = 26.90, *SD* = 8.96). Following the same criteria as Study 2a, 145 participant were excluded, which left a sample size with greater than 90% power for detecting a between-subjects effect as small as Cohen’s *d* = .22 (given the one-tailed tests specified in Study 2b’s preregistration at https://osf.io/mrty4/). Preregistering one-tailed tests meant we would only reject the null hypothesis if (a) within each condition, criterion was lower for more versus less physically attractive applicants (i.e., greater leniency toward more attractive applicants), and (b) between conditions, when an intervention either reduced criterion biases favoring more attractive applicants or increased overall sensitivity.

#### Procedure

Participants in Study 2a completed four components in the following order: Participants first received the bias-reduction intervention (if there was one); followed by the JBT, a self-report questionnaire containing measures of perceived performance, desired performance, and explicit attractiveness attitudes; and, finally, a Brief Implicit Association Test (BIAT) assessing evaluations of more and less physically attractive people. Participants in Study 2b completed components in the same order with the exception that there was no BIAT.

##### Experimental conditions

Before completing the JBT, participants were randomly assigned to a control condition that received no further instructions or one of the six interventions used in Studies 1a to 1c. Participants in the Delay intervention completed a JBT where response options did not appear until 4 s after the application first appeared. In addition, for participants in the Reward condition, a US$5 donation was made to the selected charity for each participant in the top 10% of overall accuracy.

##### JBT

Participants completed a 64-trial physical attractiveness JBT that was the same as those from prior research (e.g., Axt & Johnson, 2021; Axt & Lai, 2019). Each applicant profile contained four pieces of relevant information: Science GPA (on a scale of 1–4), Humanities GPA (on a scale of 1–4), letter of recommendation quality (with four categories: poor, fair, good, and excellent), and interview score (on a scale of 1–100). Participants were asked to accept approximately half of the applicants. Profiles were constructed such that half were more qualified and half were less qualified. Qualification was calculated by converting each piece of information to a 1 to 4 scale (GPAs retained their 4-point scale, interview scores were divided by 25, and recommendation letter quality was converted such that *poor* = 1, *fair* = 2, *good* = 3, and *excellent* = 4). More qualified applicants had a score of 14 and less qualified applicants had a score of 13. Within each level of qualification, there were an equal number of male and female White faces that were pre-rated to be more versus less physically attractive (*d* = 2.64 in Axt et al., 2018). Only White stimuli were used to remove the possible influence of race on participants’ judgment although this decision reduces the generalizability of results.

Each participant was randomly assigned to one of 12 possible JBT orders; across orders, each face was equally likely to be paired with a more versus less qualified profile.

##### Self-report measures

Participants completed three self-report items measuring perceived performance, desired performance, and explicit attractiveness preferences, each using a 7-point scale. For perceived performance, participants chose −3 when they were “extremely easier on less physically attractive applicants and tougher on more physically attractive applicants,” +3 when they were “extremely easier on more physically attractive applicants and tougher on less physically attractive applicants,” and a midpoint of 0 for “I treated both physically unattractive and physically attractive applicants equally.” The desired performance measure used the same structure but investigated how participants *wanted* to perform. Finally, for explicit attitudes, participants chose −3 when they “strongly prefer physically unattractive people to physically attractive people,” +3 when they “strongly prefer physically attractive people to physically unattractive people,” and a midpoint of 0 when they “like physically attractive and physically unattractive people equally.” See online supplemental material for full wording.

Across studies, a large majority of participants reported a perception of having been unbiased (82.9% in Study 2a, 76.9% in Study 2b) and a desire to be unbiased (93.7% in Study 2a, 85.9% in Study 2b) although explicit attitudes still showed relatively large preferences for physically attractive people (*d* = .73 in Study 2a, *d* = .77 in Study 2b).

#### BIAT

Participants in Study 1a completed a four-block, good-focal BIAT (Sriram & Greenwald, 2009) that measured evaluations toward more versus less physically attractive people. Stimuli for each attractiveness group were two male and two female faces preselected from the same images used in the JBT. Responses were analyzed using the *D* scoring algorithm (Nosek et al., 2014), with a higher score indicating more positive implicit associations toward more, versus less, physically attractive people (see online supplemental material for instructions). Study 1a participants showed a strong effect of more positive associations toward more physically attractive people (Cohen’s *d* = 1.39).

### Results

In both studies, accuracy on the JBT (accepting more qualified and rejecting less qualified applicants) was above chance performance of 50% (Study 2a: *M* = 67.8%, *SD* = 8.5; Study 2b: *M* = 66.9%, *SD* = 8.8), and levels of sensitivity were above zero (Study 2a: *M* = 1.04, *SD* = .55; Study 2b: *M* = .96, *SD* = .54). The average acceptance rate was also close to the recommended 50% (Study 2a = 51.5%; Study 2b = 52.4%).

We first conducted a series of paired samples *t* test in each condition, comparing the criterion for more versus less physically attractive applicants to investigate the presence of a response bias favoring more physically attractive applicants. In Study 2a, all conditions showed a lower criterion for more physically attractive applicants, except for the Implementation Intentions conditions, which did not show any evidence of a criterion bias (*d* = .04). In Study 2b, criterion for physically attractive applicants was significantly lower than that for less physically attractive applicants in all conditions. See Table 2 for descriptive statistics of overall JBT accuracy, sensitivity, and criterion, and see Table 3 for results of paired-samples *t* tests for criterion values within each condition.

**Table 2.** Means (and Standard Deviations) for Overall JBT Accuracy, Sensitivity, and Criterion.

Condition	Accuracy	Sensitivity	More attractive criterion	Less attractive criterion
Study 2a				
Control (*N* = 545)	67.5% (8.5)	1.01 (.54)	−.12 (.45)	.01 (.46)
Accountability (*N* = 554)	67.3% (8.9)	1.01 (.57)	−.10 (.45)	.04 (.49)
Confirmation bias (*N* = 548)	68.1% (8.5)	1.05 (.55)	−.11 (.44)	.02 (.48)
Delay (*N* = 530)	69.4% (7.7)	1.13 (.52)	−.16 (.44)	.05 (.44)
Implementation intentions (*N* = 507)	67.6% (8.9)	1.01 (.57)	−.05 (.42)	−.04 (.44)
Objectivity (*N* = 500)	67.1% (8.8)	1.00 (.56)	−.04 (.44)	.04 (.49)
Reward (*N* = 538)	67.7% (8.3)	1.04 (.54)	−.07 (.46)	.05 (.48)
Study 2b				
Control (*N* = 343)	66.3% (7.7)	0.92 (.47)	−.16 (.40)	−.004 (.47)
Accountability (*N* = 395)	65.9% (9.4)	0.90 (.56)	−.16 (.42)	.001 (.46)
Confirmation bias (*N* = 367)	66.5% (10.0)	0.95 (.62)	−.11 (.40)	−.003 (.45)
Delay (*N* = 402)	69.1% (7.8)	1.08 (.49)	−.21 (.38)	−.02 (.40)
Implementation intentions (*N* = 338)	66.4% (8.5)	0.94 (.53)	−.10 (.46)	−.03 (.44)
Objectivity (*N* = 439)	66.7% (8.2)	0.96 (.51)	−.09 (.45)	.02 (.45)
Reward (*N* = 375)	66.9% (9.3)	0.97 (.57)	−.12 (.43)	.004 (.43)

*Note*. JBT = Judgment bias task.

**Table 3.** Within-Subjects *t* Tests for Criterion Bias in Each Condition.

Condition	Test statistic	*d*	95% CI
Study 2a			
Control	*t*(544) = 5.89, *p* < .001	.25	[.17, .34]
Accountability	*t*(553) = 6.62, *p* < .001	.28	[.20, .37]
Confirmation bias	*t*(547) = 4.46, *p* < .001	.19	[.11, .27]
Delay	*t*(529) = 6.40, *p* < .001	.28	[.19, .36]
Implementation intentions	*t*(506) = 1.00, *p* = .317	.04	[−.04, .13]
Objectivity	*t*(499) = 4.20, *p* < .001	.19	[.10, .28]
Reward	*t*(537) = 6.66, *p* < .001	.29	[.20, .37]
Study 2b			
Control	*t*(342) = 6.33, *p* < .001	.34	[.23, .45]
Accountability	*t*(394) = 6.88, *p* < .001	.35	[.24, .45]
Confirmation bias	*t*(366) = 5.33, *p* < .001	.28	[.17, .38]
Delay	*t*(401) = 8.59, *p* < .001	.43	[.33, .53]
Implementation intentions	*t*(337) = 3.32, *p* = .001	.18	[.07, .29]
Objectivity	*t*(348) = 4.98, *p* < .001	.27	[.16, .37]
Reward	*t*(375) = 5.59, *p* < .001	.29	[.19, .39]

*Note*. CI = confidence interval.

Next, we conducted a series of independent samples *t* tests comparing the control condition with each experimental condition, both for overall sensitivity and for level of criterion bias (calculated as a difference score between the two values, such that higher scores indicate greater leniency toward more, versus less, physically attractive applicants). In Study 2a, criterion bias was only reduced in the Implementations Intentions condition (*d* = −.23), and sensitivity was only increased in the Delay condition (*d* = .22). Similar results were found in Study 2b, where only the Implementation Intentions condition reduced criterion bias (*d* = −.19) and only the Delay condition increased sensitivity (*d* = .33). See Table 4 for test statistics.

**Table 4.** Independent Samples *t* Tests for Criterion Bias and Sensitivity Versus Control.

Condition	Criterion bias	Sensitivity
Study 2a		
Accountability	*t*(1006) = 0.53, *p* = .598, *d* = .03, 95% CI = [−.09, .16]	*t*(1006) = −0.36, *p* = .720, *d* = −.02, 95% CI = [−.15, .10]
Confirmation Bias	*t*(998) = −1.55, *p* = .121, *d* = −.10, 95% CI = [−.22, .03]	*t*(998) = 1.29, *p* = .198, *d* = .08, 95% CI = [−.04, .21]
Delay	*t*(992) = −0.13, *p* = .900, *d* = −.01, 95% CI = [−.13, .11]	*t*(992) = 3.32, *p* = .001, *d* = .21, 95% CI = [.09, .34]
Implementation intentions	*t*(974) = −3.68, *p* < .001, *d* = −.24, 95% CI = [−.36, −.11]	*t*(974) = 0.44, *p* = .657, *d* = .03, 95% CI = [−.10, .15]
Objectivity	*t*(949) = −1.30, *p* = .192, *d* = −.08, 95% CI = [−.21, .04]	*t*(949) = 0.21, *p* = .830, *d* = .01, 95% CI = [−.11, .14]
Reward	*t*(993) = 0.47, *p* = .640, *d* = .03, 95% CI = [−.09, .15]	*t*(993) = 1.18, *p* = .239, *d* = .07, 95% CI = [−.05, .20]
Study 2b		
Accountability	*t*(736) = 0.05, *p* = .961, *d* = .004, 95% CI = [−.14, .15]	*t*(736) = −0.37, *p* = .715, *d* = −.03, 95% CI = [−.17, .12]
Confirmation bias	*t*(708) = −1.53, *p* = .126, *d* = −.12, 95% CI = [−.26, .03]	*t*(708) = 0.67, *p* = .507, *d* = .05, 95% CI = [−.10, .20]
Delay	*t*(743) = 0.96, *p* = .342, *d* = .07, 95% CI = [−.07, .21]	*t*(743) = 4.55, *p* < .001, *d* = .33, 95% CI = [.19, .48]
Implementation intentions	*t*(679) = −2.49, *p* = .013, *d* = −.19, 95% CI = [−.34, .04]	*t*(679) = 0.55, *p* = .581, *d* = .04, 95% CI = [−.11, .19]
Objectivity	*t*(690) = −1.25, *p* = .212, *d* = −.10, 95% CI = [−.24, .05]	*t*(690) = 1.08, *p* = .282, *d* = .08, 95% CI = [−.07, .23]
Reward	*t*(716) = −0.88, *p* = .381, *d* = −.07, 95% CI = [−.21, .08]	*t*(716) = 1.25, *p* = .212, *d* = .09, 95% CI = [−.05, .24]

*Note*. CI = confidence interval.

The online supplemental material has descriptive statistics for measures of implicit and explicit attitudes as well as for perceived and desired performance. A series of exploratory analyses, reported in the online supplemental material, compared whether any intervention changed perceived performance, desired performance, or explicit attitudes. No consistent results were found across studies. In Study 2a, relative to the control condition, both the confirmation bias (*d* = .19) and Delay intervention (*d* = .13) conditions were associated with increased BIAT *D* scores, showing more positive associations with physically attractive people.

#### Evaluating the accuracy of participant intuitions

There are several possible methods for evaluating the accuracy of predictions made by Studies 1a to 1c participants concerning the effectiveness of each intervention. The most stringent method would be to look at the number of participants who were “perfectly right.” Only 0.6% of participants in Study 1a, 0.9% of participants in Study 1b, and 0% of participants in Study 1c correctly guessed that only the implementation intentions and delay interventions would reduce discrimination; if anything, these rates were lower than the percentage of participants who were “perfectly wrong” in thinking that only those two interventions would *fail* to reduce discrimination (Study 1a = 2.1%, Study 1b = 2.3%, and Study 1c = 2.8%).

A second approach involves investigating accuracy for each individual intervention against chance responding (i.e., whether participants showed greater or worse accuracy than a chance responding rate of 50%). Here, a series of one-proportion *z* tests (see online supplemental material for analyses) found that, across studies, participants were above chance in predicting the (lack of) effectiveness for the reward manipulation and were above chance in predicting the actual effectiveness of the implementation intentions interventions; at the same time, participants were *below* chance in predictions about the effectiveness of the commitment to objectivity, delay, and confirmation bias interventions.

A final approach uses the rank ordering of interventions through a profile correlation analysis (Rogers et al., 2018). In this context, a profile correlation analysis can assess the degree of agreement between a participant’s ranking of intervention effectiveness and actual intervention effectiveness. To complete this analysis, data from Studies 2a and 2b were used to rank each intervention by first ranking those interventions that reliably changed either criterion bias or sensitivity (prioritizing that which produced the largest effect), and then, among interventions that did not reliably change criterion bias or sensitivity, ranking interventions by the weighted average across studies for the overall impact on criterion bias or sensitivity. This procedure produced the following ranking: (a) Delay, (b) Implementation Intentions, (c) Confirmation Bias, (d) Objectivity, (e) Accuracy Reward, and (f) Accountability. Profile correlation analyses found that, in Study 1a (*q* = −.02), Study 1b (*q* = −.02) and Study 1c (*q* = .08), the average profile correlation between each participant’s rankings and overall intervention effectiveness was essentially zero (see online supplemental material for further reporting). That is, there was no evidence that participants, on average, produced rankings of anticipated intervention effectiveness that were reliably correlated with rankings of actual effectiveness.

### Discussion

In a comparative test of six intervention strategies, two were consistently effective across studies; creating an implementation intention to avoid using faces reduced discrimination through lessening relative biases in criterion and, in a replication of prior work (Axt & Lai, 2019), requiring a delay in responding lessened discrimination through reducing the total number of errors made on the JBT. Despite the high statistical power to detect even small effects, none of the other four intervention strategies impacted JBT performance. Across several analytic approaches, there was also little evidence that participants had any insight into the collective effectiveness or ineffectiveness of these interventions.

## General Discussion

In three studies, participants reviewed six interventions meant to reduce discrimination favoring physically attractive people in a judgment task. Responses were characterized by a fair amount of confidence in at least some interventions—across studies, 61% of participants thought that at least half of the interventions would reduce discrimination—as well as by a large variability in what specific interventions would work—even the most common combination of responses was only shared among 10% of participants in Study 1a, 6.9% of participants in Study 1b, and 13.4% of participants in Study 1c.

However, in two high-powered tests of the effectiveness of these interventions, participants’ intuitions were not well-aligned with actual results. In both samples, relative to a Control condition, the Delay intervention increased overall sensitivity and the Implementation Intentions intervention reduced biases in response criterion, with the only inconsistency across samples being that the latter intervention fully debiased behavior in Study 1a but not Study 1b. Fewer than 1% of participants correctly identified the two of six interventions that actually changed JBT performance and, when evaluating each intervention individually, participants were more likely to be below than above chance in guessing whether or not each intervention would impact the magnitude of attractiveness-based discrimination. In short, participants lacked any substantive insight into how well this collection of intervention strategies would perform when deployed on novel participants.

This research speaks to ongoing efforts to address discrimination in real-life situations such as hiring, promotion, and admissions. Many nonexperts are facing renewed efforts to address socially biased discrimination within their organization, a population that has shown a continued reliance on intuition when evaluating others (e.g., Dana et al., 2013; Highhouse, 2008; Hoffman et al., 2018; Miles & Sadler-Smith, 2014). Here, we extend this work to the question of accuracy of intuitions concerning how to reduce discrimination within the specific context of physical attractiveness and, although it remains possible for intuitions to be more accurate in other domains, these results do not provide much confidence on individuals’ ability to meaningfully address discrimination when relying solely on intuition.

Practically, our results suggest that decisions made to address biases will be ineffective when not based in evidence. As a result, the data presented here can hopefully be used to convince decision-makers that it is important to look beyond one’s gut when thinking about bias reduction. Similarly, the studies also speak to a greater urgency among researchers to test interventions in more naturalistic field settings and to prioritize engaging with real-world practitioners who may be able to implement such findings.

Theoretically, these results add nuance to models of biased judgment (Fazio, 1990; Wilson & Brekke, 1994) by expanding prior work to now illustrate that, while people can often lack insight into the social information driving their biased judgment, they also lack insight into what changes will effectively limit its influence. One implication of these results is that some participants in Studies 2a and 2b may have indeed realized that their judgments favored more physically attractive applicants and, as a result, deployed a counteractive strategy based on their own intuition to correct for this influence. However, the inaccuracy in predictions from Studies 1a to 1c indicates that many intervention strategies that people believe to be effective do not actually change behavior, at least in this context. As a result, discrimination on a task such as the JBT may not only be fueled by those who fail to notice the influence of biasing information (here, physical attractiveness) but also by those who are aware of the biasing information but lack insight into what psychological processes need to occur to reduce its influence. Alerting people to their biased behavior may then be insufficient for creating change; it appears to be just as important to provide effective strategies for combating bias.

### Implications for Efforts to Reduce Discrimination

While the focus of this work is primarily on the alignment between participant intuitions and actual intervention effectiveness, Studies 2a and 2b also provide the largest comparative test of several prominent bias-reduction strategies using the same outcome measure. Despite developing six interventions based on prior academic work, only two were consistently successful at changing JBT performance; an implementation intentions strategy reduced criterion biases favoring more physically attractive applicants, and the delay intervention decreased the number of errors in which such favoritism could occur (i.e., through increased sensitivity). Although we lack definitive evidence behind why these interventions are effective, past work suggests that it is likely that participants in the Delay intervention used the extra time to better parse decision-relevant information like GPA or interview score, thereby raising the threshold for how much information needed to be gathered before a decision could be reached (Axt & Johnson, 2021).

Curiously, participants in the Implementation Intentions condition were told to ignore applicant faces, yet results found reductions in criterion biases but no changes in overall sensitivity. Given past work, these participants then looked more like those warned to avoid using physical attractiveness in their decisions, who showed reductions in criterion bias (Axt & Johnson, 2021), and less like participants who completed a blinded version of the JBT where applicant faces were removed entirely, who showed increases in overall sensitivity (Axt & Lai, 2019). This pattern suggests that, when being warned to ignore the face, participants in the Implementation Intention condition may have instead actually paid greater attention to applicant faces. As participants noticed that applicants varied on attractiveness, they may have worked to combat this bias by being more lenient toward less attractive applicants and more stringent toward more attractive applicants, resulting in no changes in the number of errors made (and therefore no differences in overall sensitivity). The Implementation Intentions intervention may have then been effective because it guided participants to attend to, and then counteract, the influence of physical attractiveness rather than ignoring attractiveness entirely, results that are consistent with other uses of this strategy, such as in reducing bias on measures of implicit associations (Lai et al., 2014; Webb et al., 2012).

However, these studies should not be taken as the final word on the relative effectiveness of any of these interventions. Aside from being constrained to a single outcome measure, many interventions may have overlooked crucial components that could have impacted performance. For instance, in the Accountability condition, participants were only warned that their behavior would be evaluated and analyzed by a group of experts. This approach may have failed to adequately heighten accountability as prior studies have also included the possibility of participants having to justify their decisions to others (Kennedy, 1993). Similarly, in the confirmation bias manipulation, participants only reviewed a 236-word explanation of confirmation bias and a reading about a potential strategy for how to reduce it. One plausible reason for the ineffectiveness of the intervention was that this manipulation did not provide enough practice at combating confirmation bias. In a prior study (Sellier et al., 2019), participants received multiple interventions to reduce confirmation bias, including a series of exercises eliciting confirmation bias and correction. Given past research on the “bias blind spot” (Pronin et al., 2002), it is possible that merely passively absorbing information about confirmation bias is insufficient to change behavior.

Finally, in the Reward condition, participants selected a charity that would receive a US$5 donation if their performance on the JBT were in the top 10% based on JBT accuracy. This more distant type of reward may then not have created enough of an incentive for participants to invest in greater cognitive resources to inhibit bias and improve accuracy. Previous work demonstrating the causal relationship between performance-contingent financial incentive and better decision-making (Stone & Ziebart, 1995; Tosi et al., 1997) all used direct payment to participants. Research on reducing discrimination will benefit from investigating whether different operationalizations of these intervention strategies will be more successful in changing behavior, either on the JBT or other outcomes, and doing so may in turn highlight the essential components needed for each intervention to be effective (e.g., Axt et al., 2019).

Alternatively, the interventions used here may have been faithful versions of each strategy, but these approaches simply do not extend to attractiveness-based discrimination. It is possible that biases based on physical attractiveness are more subtle and resistant to change than biases based on more salient social dimensions. Relatedly, a large majority of participants (80.7% across all conditions in Studies 2a and 2b) reported not thinking they used physical attractiveness in their judgments, meaning that, even if participants were committed to being objective, any enhanced objectivity may have been ineffective at changing behavior if they did not realize the influence of attractiveness on their judgments (e.g., Uhlmann & Cohen, 2007). This perspective may explain why only the Implementation Intentions intervention effectively reduced biases in criterion as it was the only intervention that explicitly named the source of the biasing social information.

In all, our studies tell us much more about the accuracy of participant intuitions when evaluating specific forms of bias-reducing interventions in a single context, and tell us much less about the overall validity of these larger intervention strategies. However, the inability to parse whether intervention effectiveness stems from an unsuccessful operationalization versus the context of physical attractiveness does not detract from our conclusions regarding the general inaccuracy of participant intuitions. All participants in Studies 1a to 1c were exposed to the exact materials that were then used in Studies 2a and 2b. Although additional tests of different versions of these interventions and across different forms of social biases will be useful, at minimum this work indicates that more basic information is needed for practitioners and researchers concerning (a) the specific characteristics that are necessary to render these intervention strategies effective, and (b) the judgment contexts in which they can be applied.

### Limitations

One potential criticism for this work concerns the exclusive focus on physical attractiveness, which, while impactful, may not be the type of discrimination that many participants think about often. It is then unclear whether the intuitions measured here would function similarly when applied to other, more salient forms of discrimination, such as pertaining to age, gender, or race. To investigate this issue, we ran a follow-up study looking at the degree to which people thought these interventions would function similarly across different social dimensions. Specifically, participants (*N* = 157) reviewed the JBT and five of the interventions used in Studies 1a to 1c,^1^ first reporting whether each would be effective at removing bias based on physical attractiveness, and then indicating whether they believed each intervention would have the same impact when the JBT was adapted to measure biases related to age, race, or gender (see online supplemental material for materials). Across all interventions, 77.7% of participants indicated that they believed each intervention would have the same impact on physical attractiveness biases as on biases related to age, gender, or race (minimum = 72.0% for the delay intervention, maximum = 82.2% for the accountability intervention).

These results indicate that, for a large majority of participants, the interventions studied here are thought to work similarly when applied to more salient forms of discrimination. At the same time, although more than 85% of Studies 2a and 2b participants reported not wanting to use physical attractiveness in their judgments, it is possible that these interventions may have produced different outcomes when applied to forms of discrimination where participants likely have even higher vigilance against appearing biased, such as for race or gender.

Another limitation may stem from our treatment of the delay intervention. Participants may not have considered lessening noise (i.e., improving overall task accuracy) as a form of discrimination reduction (Axt & Lai, 2019). Rather, participants in Studies 1a to 1c might have thought about changes to performance that were specific to how more, versus less, physically attractive people were evaluated. This is a fair critique, and one that arose due to a decision to present more straightforward instructions to participants (i.e., not having to explain the distinction between bias and noise). However, even when disregarding the delay intervention, results do not instill much confidence in the accuracy of intuition. When excluding the delay condition, 5.8% of participants across studies would have been “perfectly right” in predicting how interventions would fare at reducing discrimination, compared with 2.8% of participants who would have been “perfectly wrong.” In addition, the one-proportion *z* tests reported earlier would have still found just as many interventions to be above than below chance in accuracy when predicting the effectiveness of the five other interventions. Finally, even when only ordering all six interventions based solely on their effect on criterion biases, profile correlations showed no strong association between participant rankings and actual rankings (Study 1a *q* = .04, Study 1b *q* = .04, Study 1c *q* = .13).

A final limitation is differences among sample sources, as these studies used data from Prolific, Mechanical Turk, and Project Implicit. These differences in samples may have complicated participants’ ability to predict how various interventions would fare. However, as reported in the online supplemental material, even when only looking at our two Prolific samples (Study 1c and Study 2b), participants did not show much accuracy in their predictions regarding intervention effectiveness. For instance, one-sample *z* tests still found that Prolific participants were more likely to be below than above chance in predicting the effectiveness of each intervention on other Prolific participants. In addition, when ranking interventions based only on Study 2b results, profile correlations still showed no strong association between predicted and actual intervention effectiveness (*q* = .10). While these analyses suggest that the observed inaccuracy in intuition is not merely a function of mismatches between sample sources, it remains possible that predictions would be considerably more accurate if participants were to come from the same organization or affinity group.

Although two of the interventions were effective at reducing discrimination, the durability of this effectiveness is currently unknown, and prior work focusing on implicit associations has found that manipulations that changed performance immediately did not persist for even 24 hr (Lai et al., 2016). Understanding the durability of the implementation intentions intervention used here will be useful, especially in informing related work concerning long-term changes in prejudice (e.g., Forscher et al., 2017). Finally, as mentioned earlier, this work was constrained to one outcome measure, one type of judgment, one form of social bias, and one operationalization of each intervention strategy. Although this narrow focus does not impact our specific conclusions regarding the accuracy of participant intuitions in this context, it is possible that such accuracy could change when using other measures, when applied to other types of bias, and when tested across other versions of these interventions. Testing the generalizability of these findings is a clear avenue for subsequent work.

### Future Directions

This research program may benefit from further exploration of two related questions. First, these studies did not collect any information about participant confidence in Studies 1a to 1c. It is unclear whether the predictions made about intervention effectiveness were highly confident (and largely inaccurate) or were merely reflecting participants’ “best guess,” meaning participants would not have then been overly surprised to learn about how often they were wrong in their responses. Prior work on more factual judgments suggests that participants are typically overconfident in their predictions, but this overconfidence can vary substantially across domains (e.g., Soll & Klayman, 2004), and the role of overconfidence in predictions about bias-reducing interventions is currently unknown. Greater knowledge of the role of participant confidence will also shed light on the real-world implications of this issue.

Second, these samples were limited to individuals without expertise in research on biased judgment. Prior work has found that experts have some accuracy in predicting the outcomes of both replication studies (Dreber et al., 2015; Forsell et al., 2019) and novel data collections (DellaVigna & Pope, 2018), and the same may be true of experts when predicting the relative success of the interventions used here, although the fact that each of these interventions was based on the success of prior work may lead even experts to suffer from the same levels of inaccuracy as exhibited by nonexpert participants. Regardless, an expert sample of either researchers familiar with these strategies or professionals with significant hiring or admissions experience will be informative for understanding whether insight into the effectiveness of interventions can be developed over time. Similarly, it is possible that participants who were more likely to have been the target of discrimination, either through identifying as being low in physical attractiveness or through having another stigmatized identity, may have greater insight into which of these interventions would most effectively reduce discrimination on the JBT.

## Conclusion

Increased calls to address discrimination within organizations will lead to the implementation of new interventions and changes, a push that is complicated by a general lack of evidence-based methods for addressing bias and by a widespread faith in intuition when evaluating others. Results of two studies testing six different manipulations found that a pair of interventions—rehearsing distraction-inhibiting if-then statements and requiring a delay in responses—significantly reduced the magnitude of attractiveness-based discrimination on a judgment task, although these results were poorly aligned with predictions about how to address such discrimination.

Aside from being the largest comparative assessment to date of strategies to reduce biased judgment, this work reveals that, just as people can often have little insight into the social information influencing their behavior, this lack of insight extends into strategies for minimizing biased behavior. This work shows that addressing bias will take more than good intentions and strong feelings, and results only further stress the need for the creation and adoption of effective, evidence-based practices in reducing discrimination.

## Supplemental Material

sj-docx-1-psp-10.1177_01461672221074748 – Supplemental material for Misplaced Intuitions in Interventions to Reduce Attractiveness-Based Discrimination Click here for additional data file. Supplemental material, sj-docx-1-psp-10.1177_01461672221074748 for Misplaced Intuitions in Interventions to Reduce Attractiveness-Based Discrimination by Jordan R. Axt, Juanyu Yang and Harshadaa Deshpande in Personality and Social Psychology Bulletin

## Notes

1. We removed the implementation intentions intervention as the manipulation specifically mentions applicant faces and, as a result, may have confused participants when asked whether it would generalize to other forms of social biases.
