# Supplementary material for: Misplaced Intuitions in Interventions to Reduce Attractiveness-Based Discrimination
Source: Pers Soc Psychol Bull. 2022 Feb 18;49(4):527–40. doi: 10.1177/01461672221074748 (PMC9989229; doi:10.1177/01461672221074748)
Supplement: sj-docx-1-psp-10.1177_01461672221074748 – Supplemental material for Misplaced Intuitions in Interventions to Reduce Attractiveness-Based Discrimination [file sj-docx-1-psp-10.1177_01461672221074748.docx]

**Online Supplement:** Misplaced Intuitions in Interventions to Reduce Discrimination

Study 1a-1c: Wording and Stimuli ……………………………………………………..……..... 2

Study 1a-1c: Results of Wilcoxan Sign-Rank Tests ………...………………………..…...….... 10

Study 2a & 2b: Self-Report Items ……...……………………………………….……...………. 12

Study 2a: BIAT Instructions …………………………………………………………...………. 13

Study 2a & 2b: Implicit/Explicit Attitudes, Perceived Performance, Desired Performance….... 14

Study 2a & 2b: One-Proportion *Z* Tests Against Chance Responding…………..……...…….... 17

Study 1a-1c: Profile Correlation Analysis: Full Reporting………………………...………….... 18

General Discussion Study: Materials ………………………………………………...……….... 19

Analysis Among Only Prolific Users ………………………………...……………...……….... 22

**Study 1a-1c: Full Wording and Stimuli**

*JBT Introduction*

In this task, participants are told that they are on the selection committee for an academic honour society. Participants will see the profiles of 64 applicants. Each applicant's profile is presented with four pieces of relevant information; their science GPA, humanities GPA, letters of recommendation and interview score. Participants are asked to weigh the four pieces of information equally and try to select the most qualified applicants. Participants are asked to accept around half the applicants.

In the task, some applicants are objectively more qualified and should be accepted while others are objectively less qualified and should be rejected. At the same time, each applicant's profile is paired with a face that is more or less physically attractive. Both the more and less qualified applications are split evenly to have equal numbers of more and less physically attractive faces, meaning attractiveness cannot be used to determine whether or not an applicant is qualified.

On the next pages, you will see some sample applicants in the task. Please note you only need to review the profiles, you do NOT need to accept or reject any applicants. A button to move to the next page will appear after 5 seconds.

*JBT Sample Stimuli*


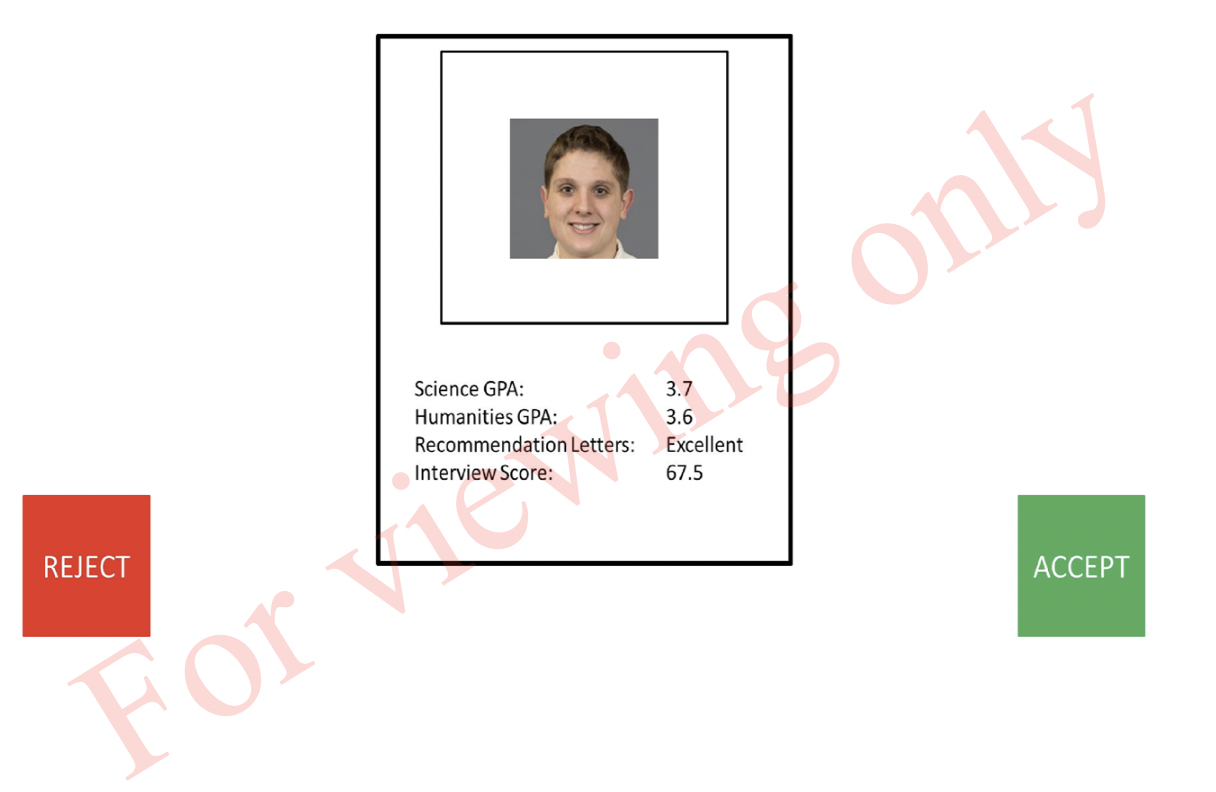


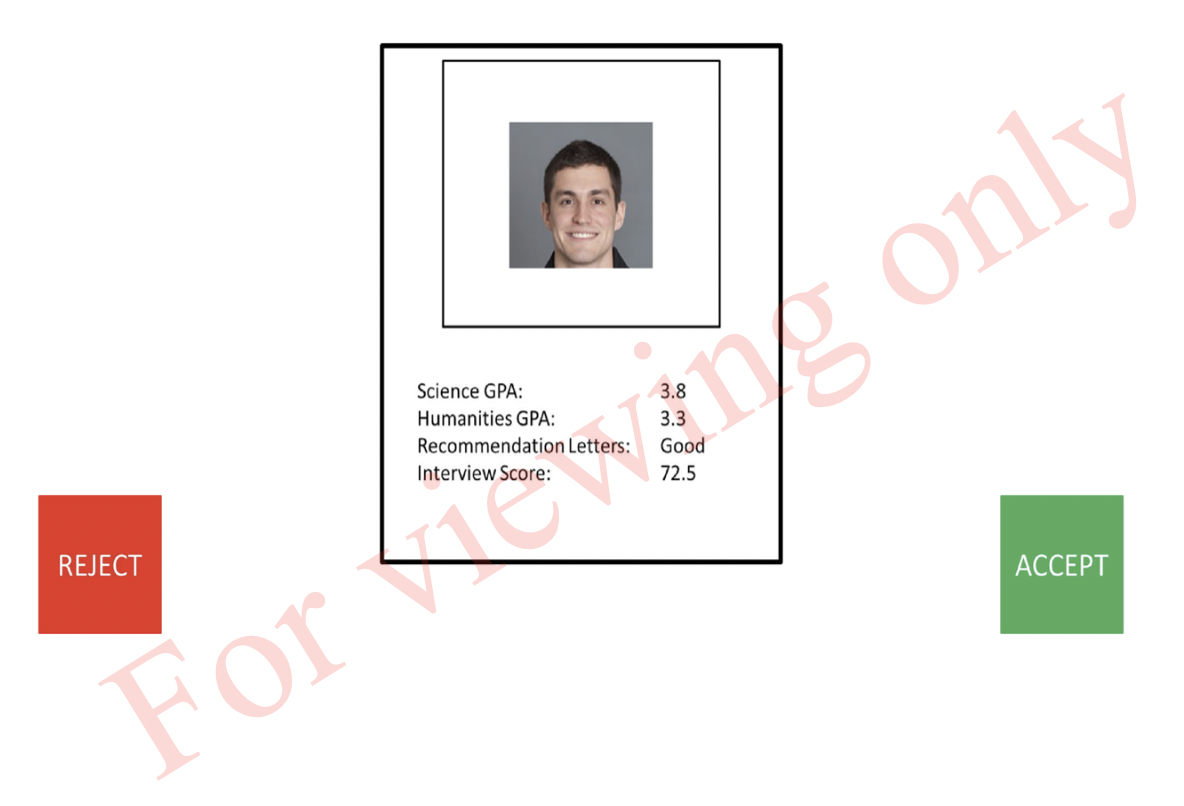


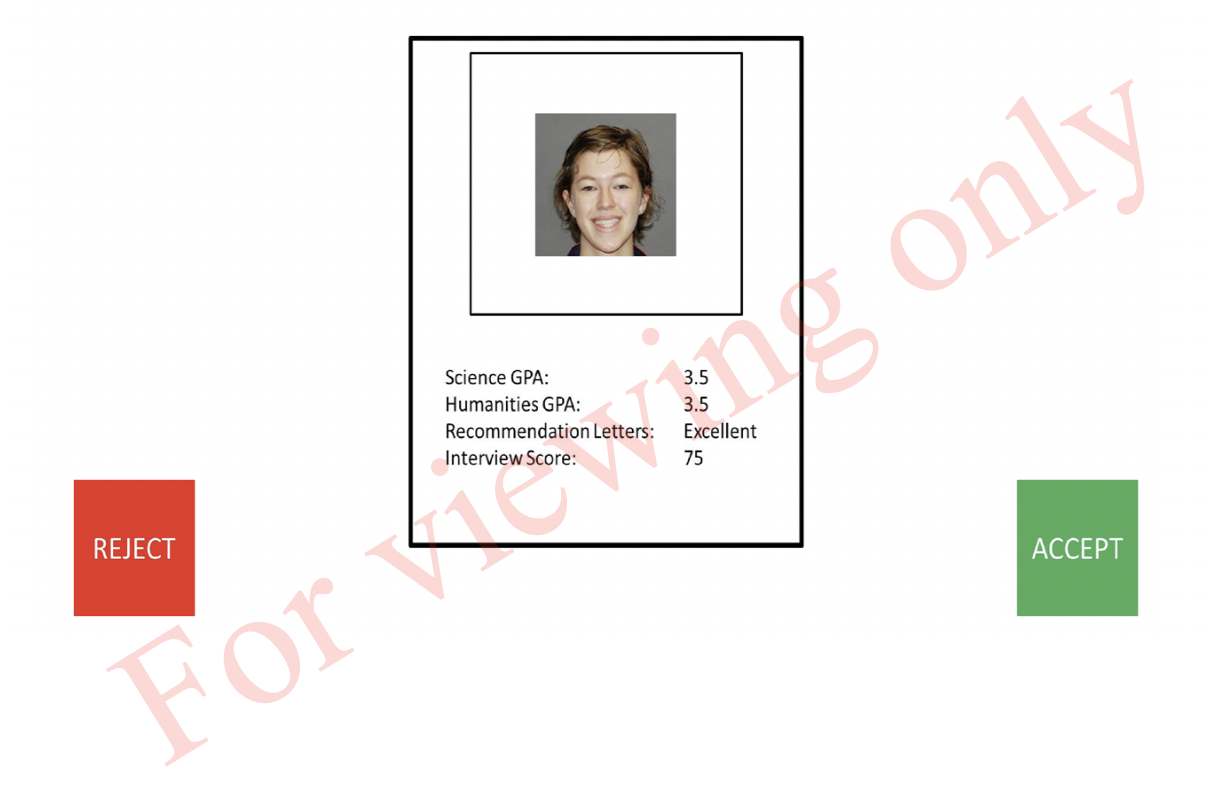


*Summary of Typical JBT Performance*

People that complete this task typically show a bias favouring more physically attractive applicants. This means that despite being equally qualified, people go easier on more relative to less physically attractive applicants. On average, a physically attractive applicant is about 9% more likely to be accepted into the honour society than a less physically attractive applicant.

*Introduction to Interventions*

[Page 1]

Researchers are soon going to test a variety of interventions that may reduce this attractiveness bias. On the following screens you will be shown the **six** interventions that are about to be tested to try and reduce this bias favouring more physically attractive people.

Specifically, each participant will see one of the **six** possible interventions right before they start evaluating applicants to the honour society and seeing the more versus less physically attractive faces.

We are interested in getting your own thoughts on what intervention you think will best reduce this bias favouring more physically attractive people. What will be the most effective? What will be the least effective?

[Page 2]

There will be six total interventions: Increasing Accountability, Committing to Objectivity, Making A Strategy, Learning About Biased Judgment, Slowing Down, Rewarding Accuracy.

Please read each intervention carefully and at the end, you will be asked to rank them based on how effective YOU think they will be at reducing the impact of physical attractiveness on participants’ decision-making.

On each page, you will see an image of the intervention that will be shown to participants. Please look over the intervention for at least 20 seconds. After 20 seconds, a button will appear that allows you to proceed to view the next intervention.

Accountability Intervention

In this study you will be asked to imagine you are on the selection committee for an academic honor society. During the task, you will decide whether to accept or reject a number of applicants.

It is important to note that your decisions on the task will be reviewed by a panel of researchers that are experienced in evaluating students. They will analyze your performance in terms of accurately accepting more qualified applicants and rejecting less qualified applicants.

Objectivity Intervention

In this study you will be asked to imagine you are on the selection committee for an academic honor society. During the task, you will decide whether to accept or reject a number of applicants.

Studies have shown that although people want to be accurate on the task, they often show behavior that is inconsistent with this goal.

In order to meet this goal of being more accurate, it may be helpful for you to adopt an objective mindset. This can be accomplished by deciding on a set of parameters you will use to evaluate students **before** beginning the task. Committing to such a strategy beforehand is important because it could prevent you from being distracted by irrelevant information during the task.

As a reminder, below is a sample of the types of criteria you will see in the task:

Science GPA: 3.7

Humanities GPA: 3.6

Recommendation Letters: Excellent

Interview Score: 67.5

Before you being the task, we want to know more about how you will objectively evaluate each application. Please answer the following questions in the space provided below.

**Q1: What information will you consider when evaluating applicants?**

**Q2: Why is this information relevant to your evaluation?**

Implementation Intentions Intervention

In this study you will be asked to imagine you are on the selection committee for an academic honor society. During the task, you will decide whether to accept or reject a number of applicants.

In order to select the most qualified applicants, you should be careful to not let irrelevant information affect your decisions. In order to help you achieve this, research has shown it to be helpful for you to adopt the following strategy: “*If I see a student’s application, then I will ignore their face”*.

Please mentally repeat this strategy three times using inner speech. When you are comfortable recalling it, click the arrow to go to the next page.

Please type out the strategy you learned on the previous page in the box below.

Confirmation Bias Intervention

In this study you will be asked to imagine you are on the selection committee for an academic honor society. During the task, you will decide whether to accept or reject a number of applicants.

Research has shown that in order to be accurate on the task, it is important to reduce one’s susceptibility to something called “confirmation bias”. Confirmation bias occurs when someone selectively searches for information that validates their hypothesis and ignores information that may be inconsistent with their beliefs. As a result, confirmation bias means that people will ignore other important information that might indicate that their hypothesis is, in fact, incorrect. For example, an individual with a fear of flying in airplanes is more likely to search the internet for news reports about airplane accidents while ignoring robust data suggesting that airplanes are quite safe.

Confirmation bias can affect an individual’s decisions on the academic selection task. For example, under confirmation bias, if one believes a student to be qualified, they might focus on the student’s high interview score while ignoring their low GPA and low recommendation letter strength. Alternatively, if one believes a student to be unqualified, they might focus on the student’s low GPA while ignoring their high interview score and recommendation letter strength.

In order to overcome confirmation bias, research has shown it to be helpful to adopt a “hypothesis disconfirming” strategy. You can adopt this strategy by looking for information that suggests a student may be unqualified, rather than only looking for information that suggests a student may be qualified. Adopting this strategy will make it easier to eliminate students that are unqualified.

Delay Intervention

In this study you will be asked to imagine you are on the selection committee for an academic honor society. During the task, you will decide whether to accept or reject a number of applicants.

Please read carefully:

Past research suggests that people may be more accurate at evaluating applicants if they can slow down and spend more time reflecting on each decision. To help you do so, there will be a four second delay between when the application is first presented and when you will be able to make an accept or reject decision. You can use those four seconds to think more about your decisions.

Reward Intervention

In this study you will be asked to imagine you are on the selection committee for an academic honor society. During the task, you will decide whether to accept or reject a number of applicants.

We are interested in your ability to accept the more qualified applicants and reject the less qualified applicants. To motivate you to perform well, participants who are in the top 10% for accuracy will have a $5 donation made to a charity of their choice. Below is a list of fifteen charities that have earned an ‘A’ or higher grade from the independent website charitywatch.org.

Please select the charity that you would like to receive a donation if you are in the top 10% in terms of accuracy on the selection task:

Bowery Residents’ Committee (BRC)

Brain & Behavior Research Foundation

Breast Cancer Research Foundation

Children’s Defense Fund

Compassion International

Elizabeth Glaser Pediatric AIDS Foundation

Farm Aid

Fisher House Foundation

Food and Water Watch

Goodwill Industries International

Helen Keller International

International Peace Institute

Lupus Research Alliance

Scholarship America

Wildlife Conservation Society

Ranking Intervention Item

**Now that you have read about each of the six interventions, we are interested in your thoughts on what will be most effective at reducing the physical attractiveness bias.**

Listed below are the six interventions described on previous slides. Please rank the interventions from ‘1’ to ‘6’ based on YOUR perception of how effective they will be in reducing discrimination, where ‘1’ is **most** effective and ‘6’ is **least** effective. You can rank the interventions by dragging them around the screen.

- **Increasing accountability** – Learning that decisions will be reviewed by a panel of researchers

- **Commitment to objectivity** – Focusing on relevant information while evaluating applicants

- **Making a strategy** – Adopting a strategy of ‘If I see a student, then I will ignore their face’

- **Learning about biased judgment** – Reading about confirmation bias and how to counter it

- **Slowing down** – Each judgment is delayed four seconds

- **Rewarding Accuracy** – Best performers get to donate to charity of their choice

Effective at All Item

**Aside from ranking the interventions, we are interested in your thoughts on which interventions will be effective in reducing the physical attractiveness bias at all.**

Below, select each of the interventions that you believe will reduce the physical attractiveness bias. You can select as many or as few interventions as you would like. For any intervention you do not select, it means you think it will be ineffective at reducing the physical attractiveness bias.

- I believe that **none** of the applications will reduce physical attractiveness bias [only include in Study 1b]

- **Increasing accountability** – Learning that decisions will be reviewed by a panel of researchers

- **Commitment to objectivity** – Focusing on relevant information while evaluating applicants

- **Making a strategy** – Adopting a strategy of ‘If I see a student, then I will ignore their face’

- **Learning about biased judgment** – Reading about confirmation bias and how to counter it

- **Slowing down** – Each judgment is delayed four seconds

- **Rewarding Accuracy** – Best performers get to donate to charity of their choice

Others’ Perceptions Item (Removed in Study 1c)

**We also want your thoughts on what OTHER PEOPLE will think are the most and least effective interventions.**

Please rank the interventions from ‘1’ to ‘6’ based on how effective you think OTHER PEOPLE might consider them to be, where ‘1’ is **most** effective and ‘6’ is **least** effective. You can rank the interventions by dragging them around the screen.

- **Increasing accountability** – Learning that decisions will be reviewed by a panel of researchers

- **Commitment to objectivity** – Focusing on relevant information while evaluating applicants

- **Making a strategy** – Adopting a strategy of ‘If I see a student, then I will ignore their face’

- **Learning about biased judgment** – Reading about confirmation bias and how to counter it

- **Slowing down** – Each judgment is delayed four seconds

- **Rewarding Accuracy** – Best performers get to donate to charity of their choice

**Study 1a-1c: Wilcoxon Sign-Rank Results**

Study 1a

*Descriptive Statistics for Intervention Rank*

Accountability: *M* = 3.34, *SD* = 1.60

Objectivity: *M* = 2.84, *SD* = 1.59

Implementation Intentions: *M* = 3.04, *SD* = 1.68

Confirmation Bias: *M* = 3.20, *SD* = 1.53

Delay: *M* = 4.24, *SD* = 1.59

Accuracy: *M* = 4.33, *SD* = 1.67

*Wilcoxon Sign-Rank Comparisons*

Objectivity – Implementation Intentions: *Z* = 1.56, *p* =.119

Objectivity – Confirmation Bias: *Z* = 2.85, *p* = .004

Implementation Intentions – Confirmation Bias: *Z* = .99, *p* = .321

Implementation Intentions – Accountability: *Z* = 2.15, *p* = .032

Confirmation Bias – Accountability: *Z* = 1.30, *p* = .303

Confirmation Bias – Delay: *Z* = 7.23, *p* < .001

Accountability – Delay: *Z* = 6.07, *p* < .001

Delay – Accuracy: *Z* = .66, *p* = .507

Study 1b

*Descriptive Statistics for Intervention Rank*

Accountability: *M* = 3.42, *SD* = 1.60

Objectivity: *M* = 2.66, *SD* = 1.52

Implementation Intentions: *M* = 3.13, *SD* = 1.67

Confirmation Bias: *M* = 3.23, *SD* = 1.61

Delay: *M* = 4.24, *SD* = 1.58

Accuracy: *M* = 4.33, *SD* = 1.62

Objectivity – Implementation Intentions: *Z* = 3.51, *p* <.001

Implementation Intentions – Confirmation Bias: *Z* = .62, *p* =.538

Implementation Intentions – Accountability: *Z* = 2.19, *p* =.028

Confirmation Bias – Accountability: *Z* = 1.40, *p* =.163

Confirmation Bias – Delay: *Z* = 7.01, *p* <.001

Accountability – Delay: *Z* = 5.61, *p* <.001

Delay – Accuracy: *Z* = .72, *p* =.469

Study 1c

*Descriptive Statistics for Intervention Rank*

Accountability: *M* = 3.40, *SD* = 1.42

Objectivity: *M* = 2.49, *SD* = 1.49

Implementation Intentions: *M* = 3.18, *SD* = 1.69

Confirmation Bias: *M* = 2.98, *SD* = 1.54

Delay: *M* = 4.13, *SD* = 1.54

Accuracy: *M* = 4.81, *SD* = 1.49

*Wilcoxon Sign-Rank Comparisons*

Objectivity – Implementation Intentions: *Z* = 4.24, *p* <.001

Objectivity – Confirmation Bias: *Z* = 3.00, *p* = .003

Implementation Intentions – Confirmation Bias: *Z* = .98, *p* = .328

Implementation Intentions – Accountability: *Z* = 1.35, *p* = .178

Confirmation Bias – Accountability: *Z* = 2.88, *p* = .004

Confirmation Bias – Delay: *Z* = 6.89, *p* < .001

Accountability – Delay: *Z* = 4.08, *p* < .001

Delay – Accuracy: *Z* = 4.10, *p* < .001

**Study 2a & Study2b: Self-Reported Items**

Which statement best describes your **performance** on the task?

I was extremely easier on physically unattractive applicants and tougher on physically attractive applicants,

I was moderately easier on physically unattractive applicants and tougher on physically attractive applicants,

I was slightly easier on physically unattractive applicants and tougher on physically attractive applicants,

I treated both physically unattractive and physically attractive applicants equally',

I was slightly easier on physically attractive applicants and tougher on physically unattractive applicants,

I was moderately easier on physically attractive applicants and tougher on physically unattractive applicants,

I was extremely easier on physically attractive applicants and tougher on physically unattractive applicants

Which statement best describes how you **wanted** to perform on the task?

I wanted to be extremely easier on physically unattractive applicants and tougher on physically attractive applicants',

I wanted to be moderately easier on physically unattractive applicants and tougher on physically attractive applicants',

I wanted to be slightly easier on physically unattractive applicants and tougher on physically attractive applicants',

I wanted to treat both physically unattractive and physically attractive applicants equally',

I wanted to be slightly easier on physically attractive applicants and tougher on physically

unattractive applicants',

I wanted to be moderately easier on physically attractive applicants and tougher on

physically unattractive applicants',

I wanted to be extremely easier on physically attractive applicants and tougher on

physically unattractive applicants'

Which statement best describes you?

I strongly prefer physically attractive people to physically unattractive people',

I moderately prefer physically attractive people to physically unattractive people',

I slightly prefer physically attractive people to physically unattractive people',

I like physically attractive people and physically unattractive people equally',

I slightly prefer physically unattractive people to physically attractive people',

I moderately prefer physically unattractive people to physically attractive people',

I strongly prefer physically unattractive people to physically attractive people'

**Study 2a: BIAT Instructions**


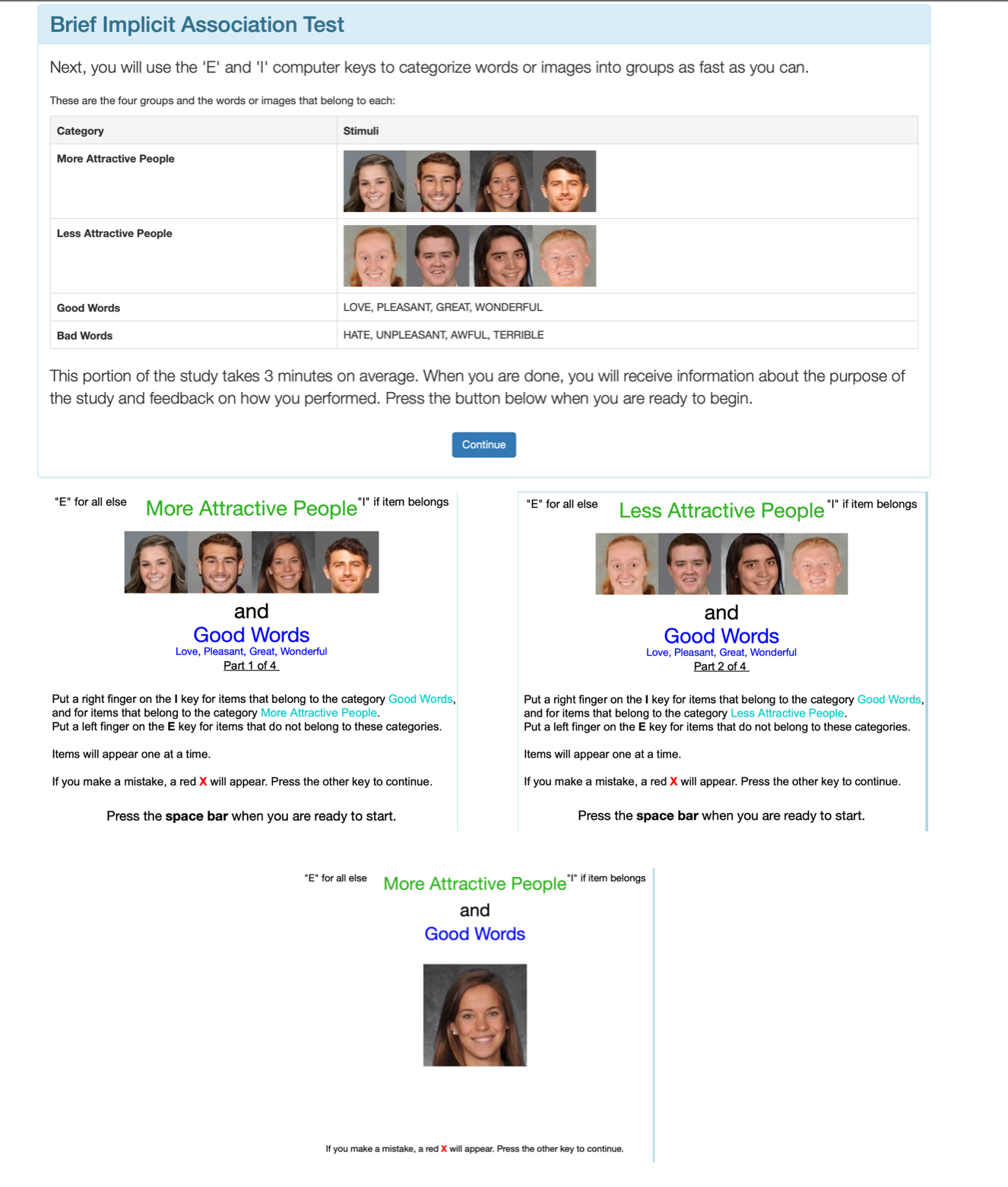


**Study 2a & 2b: Implicit/Explicit Attitudes, Perceived Performance, Desired Performance**

Implicit Attitudes:

*Study 2a*

Control: *M* = .65, *SD* = .47

Accountability: *M* = .66, *SD* = .52

Confirmation Bias: *M* = .75, *SD* = .47

Delay: *M* = .72, *SD* = .51

Implementation Intentions: *M* = .64, *SD* = .51

Objectivity: *M* = .68, *SD* = .48

Reward: *M* = .69, *SD* = .49

Control vs. Accountability: *t*(995) = .15, *p* = .884

Control vs. Confirmation Bias: *t*(993) = 3.12, *p* = .002

Control vs. Delay: *t*(986) = 2.09, *p* = .037

Control vs. Implementation Intentions: *t*(968) = .30, *p* = .761

Control vs. Objectivity: *t*(945) = .93, *p* = .353

Control vs. Reward: *t*(990) = 1.20, *p* = .232

Explicit Attitudes

*Study 2a*

Control: *M* = .62, *SD* = .82

Accountability: *M* = .62, *SD* = .91

Confirmation Bias: *M* = .69, *SD* = .87

Delay: *M* = .61, *SD* = .90

Implementation Intentions: *M* = .66, *SD* = .87

Objectivity: *M* = .64, *SD* = .87

Reward: *M* = .63, *SD* = .89

Control vs. Accountability: *t*(1057) = 0.08, *p* = .938

Control vs. Confirmation Bias: *t*(1051) = 1.49, *p* = .138

Control vs. Delay: *t*(1037) = .20, *p* = .843

Control vs. Implementation Intentions: *t*(1010) = .82, *p* = .414

Control vs. Objectivity: *t*(1005) = .44, *p* = .661

Control vs. Reward: *t*(1042) = .20, *p* = .843

*Study 2b*

Control: *M* = .74, *SD* = 1.00

Accountability: *M* = .78, *SD* = 1.04

Confirmation Bias: *M* = .84, *SD* = 1.02

Delay: *M* = .76, *SD* = .97

Implementation Intentions: *M* = .83, *SD* = 1.01

Objectivity: *M* = .71, *SD* = .99

Reward: *M* = .72, *SD* = .94

Control vs. Accountability: *t*(730) = .53, *p* = .596

Control vs. Confirmation Bias: *t*(702) = 1.31, *p* = .190

Control vs. Delay: *t*(735) = .28, *p* = .779

Control vs. Implementation Intentions: *t*(671) = 1.28, *p* = .199

Control vs. Objectivity: *t*(683) = .29, *p* = .769

Control vs. Reward: *t*(710) = .28, *p* = .780

Perceived Performance

*Study 2a*

Control: *M* = .10, *SD* = .60

Accountability: *M* = .08, *SD* = .73

Confirmation Bias: *M* = .05, *SD* = .54

Delay: *M* = .11, *SD* = .57

Implementation Intentions: *M* = .04, *SD* = .54

Objectivity: *M* = .12, *SD* = .59

Reward: *M* = .07, *SD* = .57

Control vs. Accountability: *t*(1070) = .64, *p* = .524

Control vs. Confirmation Bias: *t*(1061) = 1.51, *p* = .133

Control vs. Delay: *t*(1050) = .11, *p* = .914

Control vs. Implementation Intentions: *t*(1019) = 1.59, *p* = .113

Control vs. Objectivity: *t*(1014) = .37, *p* = .711

Control vs. Reward: *t*(1057) = .78, *p* = .438

*Study 2b*

Control: *M* = .11, *SD* = .82

Accountability: *M* = .08, *SD* = .80

Confirmation Bias: *M* = -.03, *SD* = .80

Delay: *M* = .11, *SD* = .79

Implementation Intentions: *M* = -.03, *SD* = .79

Objectivity: *M* = -.01, *SD* = .76

Reward: *M* = .06, *SD* = .75

Control vs. Accountability: *t*(732) = .59, *p* = .553

Control vs. Confirmation Bias: *t*(704) = 2.27, *p* = .023

Control vs. Delay: *t*(734) = .05, *p* = .958

Control vs. Implementation Intentions: *t*(671) = 2.28, *p* = .023

Control vs. Objectivity: *t*(685) = 2.09, *p* = .037

Control vs. Reward: *t*(712) = .90, *p* = .368

Desired Performance

*Study 2a*

Control: *M* = 0, *SD* = .39

Accountability: *M* = -.02, *SD* = .41

Confirmation Bias: *M* = -.02, *SD* = .37

Delay: *M* = -.004, *SD* = 41

Implementation Intentions: *M* = -.01, *SD* = .35

Objectivity: *M* = -.004, *SD* = .39

Reward: *M* = -.02, *SD* = .38

Control vs. Accountability: *t*(1057) = .92, *p* = .359

Control vs. Confirmation Bias: *t*(1047) = .73, *p* = .467

Control vs. Delay: *t*(1045) = .16, *p* = .876

Control vs. Implementation Intentions: *t*(1009) = .35, *p* = .726

Control vs. Objectivity: *t*(1005) = .17, *p* = .867

Control vs. Reward: *t*(1039) = .90, *p* = .370

*Study 2b*

Control: *M* = .04, *SD* = .67

Accountability: *M* = -.05, *SD* = .66

Confirmation Bias: *M* = -.07, *SD* = .69

Delay: *M* = -.003, *SD* = .65

Implementation Intentions: *M* = .03, *SD* = .55

Objectivity: *M* = -.02, *SD* = .65

Reward: *M* = -.07, *SD* = .63

Control vs. Accountability: *t*(727) = 1.93, *p* = .054

Control vs. Confirmation Bias: *t*(696) = 2.26, *p* = .024

Control vs. Delay: *t*(734) = .96, *p* = .337

Control vs. Implementation Intentions: *t*(664) = .29, *p* = .771

Control vs. Objectivity: *t*(678) = 1.28, *p* = .201

Control vs. Reward: *t*(704) = 2.29, *p* = .022

**One-Proportion *Z* Tests Against Chance Responding**

Accountability Intervention

Across Studies 1a-1c, 430 of 902 participants (47.7%) thought the accountability intervention would be effective. Given that Studies 2a-2b found no evidence that the accountability intervention impacted performance, a one-proportion *Z* test against 50% found a value of *X*^2^ (1, *N* = 902) = 1.40, *p* = .167, meaning that performance did not reliably differ from chance.

Objectivity Intervention

Across Studies 1a-1c, 597 of 902 participants (66.3%) thought the objectivity intervention would be effective. Given that Studies 2a-2b found no evidence that the objectivity intervention impacted performance, a one-proportion *Z* test against 50% found a value of *X*^2^ (1, *N* = 902) = 9.80, *p* < .001, meaning that performance was reliably *below* chance.

Implementation Intentions Intervention

Across Studies 1a-1c, 524 of 902 participants (58.2%) thought the implementation intentions intervention would be effective. Given that Studies 2a-2b found consistent evidence that the implementation intentions intervention impacted performance, a one-proportion *Z* test against 50% found a value of *X*^2^ (1, *N* = 902) = 4.90, *p* < .001, meaning that performance was reliably *above* chance.

Confirmation Bias

Across Studies 1a-1c, 502 of 902 participants (55.6%) thought the confirmation bias intervention would be effective. Given that Studies 2a-2b found no evidence that the confirmation bias intervention impacted performance, a one-proportion *Z* test against 50% found a value of *X*^2^ (1, *N* = 902) = 3.40, *p* < .001, meaning that performance was reliably *below* chance.

Delay

Across Studies 1a-1c, 263 of 902 participants (29.2%) thought the response delay intervention would be effective. Given that Studies 2a-2b found consistent evidence that the delay intervention impacted performance, a one-proportion *Z* test against 50% found a value of *X*^2^ (1, *N* = 902) = 12.50, *p* < .001, meaning that performance was reliably *below* chance.

Accuracy Reward

Across Studies 1a-1c, 240 of 902 participants (26.6%) thought the accuracy reward intervention would be effective. Given that Studies 2a-2b found no evidence that the reward intervention impacted performance, a one-proportion *Z* test against 50% found a value of *X*^2^ (1, *N* = 902) = 14.0, *p* < .001, meaning that performance was reliably *above* chance.

**Profile Correlation Analysis: Full Reporting**

In Study 1a, a profile correlation analysis was run among all 338 eligible participants, assessing the correlation between each participant’s ranking of intervention effectiveness and the actual ranking of intervention effectiveness (see main text for procedure to determine ranking). Using the R code provided by Rogers, Wood and Furr (2018), the average profile correlation value was *q* = -.024 (median: *q* = -.029, 1^st^ quartile: *q* = -.31, 3^rd^ Quartile: *q* = .37). The average correlation, being both negative and very close to zero, indicates no substantive relationship between participant effectiveness rankings and rankings of actual intervention effectiveness.

This analysis was repeated among the 349 eligible participants in Study 1b. Here, the average profile correlation value was *q* = -.019 (median: *q* = -.029, 1^st^ quartile: *q* = -.37, 3^rd^ Quartile: *q* = .37). Finally, for the 215 eligible participants in Study 1c, the average profile correlation value was *q* = .078 (median: *q* = .086, 1^st^ quartile: *q* = -.257, 3^rd^ Quartile: *q* = .429). Again, these average correlation values being close to zero indicates no substantive relationship between participant effectiveness rankings and rankings of actual intervention effectiveness.

**General Discussion Study: Materials**

Participants first viewed the same materials as participants in Studies 1a-1b, though the implementation intentions intervention was removed. This study also removed the items related to predicting how other participants would rank the effectiveness of the interventions. Next, participants viewed the following text introducing the idea of whether these interventions would work similarly in other domains:

**Now imagine that the decision-making task was adapted to look at social biases other than physical attractiveness. Specifically, imagine that separate versions of the task were run to investigate biases related to age, gender, and race.**

Do you think the interventions you learned about here would have the same effect on attractiveness biases as they do on other forms of social bias? For instance, you may believe that rewarding accuracy will fail to reduce biases based on physical attractiveness and would also be ineffective at reducing biases based on age, gender, or race.

Or will the interventions have different effects on other forms of biases? For instance, you may believe that slowing down will do nothing to impact physical attractiveness biases, but could effectively combat biases related to either age, gender, or race.

For the final part of this study, you will go through each of the five interventions and tell us whether you think it will have a same or different impact on these other types of biases as you thought it would on the attractiveness bias.

After reading this text, participants completed an item for each intervention asking them to report whether that intervention would have the same or different effects when applied to other contexts. Items were presented in a randomized order and are reported in full below.

1. Think about the **Increasing Accountability intervention** (Learning that decisions will be reviewed by a panel of researchers).

Will this intervention have the same impact (or lack of an impact) when applied to either age, gender, or race biases?

I believe the **Increasing Accountability** intervention will have the **SAME** impact on attractiveness bias as it would on biases related to age, gender, or race.

I believe the **Increasing Accountability** intervention will have a **DIFFERENT** impact on attractiveness bias as it would on biases related to age, gender, or race.

2. Think about the **Commitment to Objectivity intervention** (Focusing on relevant information while evaluating applicants).

Will this intervention have the same impact (or lack of an impact) when applied to either age, gender, or race biases?

I believe the **Commitment to Objectivity** intervention will have the **SAME** impact on attractiveness bias as it would on biases related to age, gender, or race.

I believe the **Commitment to Objectivity** intervention will have a **DIFFERENT** impact on attractiveness bias as it would on biases related to age, gender, or race.

3. Think about the **Learning About Biased Judgement intervention**(Reading about confirmation bias and how to counter it).

Will this intervention have the same impact (or lack of an impact) when applied to either age, gender, or race biases?

I believe the **Learning About Biased Judgment** intervention will have the **SAME** impact on attractiveness bias as it would on biases related to age, gender, or race.

I believe the **Learning About Biased Judgment** intervention will have a **DIFFERENT** impact on attractiveness bias as it would on biases related to age, gender, or race.

4. Think about the **Slowing Down intervention**(Each judgment is delayed four seconds).

Will this intervention have the same impact (or lack of an impact) when applied to either age, gender, or race biases?

I believe the **Slowing Down** intervention will have the **SAME** impact on attractiveness bias as it would on biases related to age, gender, or race.

I believe the **Slowing Down** intervention will have a **DIFFERENT** impact on attractiveness bias as it would on biases related to age, gender, or race.

5. Think about the **Rewarding Accuracy intervention**(Best performers get to donate to a charity of their choice).

Will this intervention have the same impact (or lack of an impact) when applied to either age, gender, or race biases?

I believe the **Rewarding Accuracy** intervention will have the **SAME** impact on attractiveness bias as it would on biases related to age, gender, or race.

I believe the **Rewarding Accuracy** intervention will have a **DIFFERENT** impact on attractiveness bias as it would on biases related to age, gender, or race.

**Analysis Among Only Prolific Participants**

**One-Sample *Z*-Tests**

Accountability Intervention

In Study 1c, 104 of 214 participants (48.6%) thought the accountability intervention would be effective. Given that Study 2b found no evidence that the accountability intervention impacted performance, a one-proportion *Z* test against 50% found a value of *X*^2^ (1, *N* = 214) = .40, *p* = .682, meaning that performance did not reliably differ from chance.

Objectivity Intervention

In Study 1c, 171 of 214 participants (79.9%) thought the objectivity intervention would be effective. Given that Study 2b found no evidence that the objectivity intervention impacted performance, a one-proportion *Z* test against 50% found a value of *X*^2^ (1, *N* = 214) = 8.70, *p* < .001, meaning that performance was reliably *below* chance.

Implementation Intentions Intervention

In Study 1c, 128 of 214 participants (59.8%) thought the implementation intentions intervention would be effective. Given that Study 2b found evidence that the implementation intentions intervention impacted performance, a one-proportion *Z* test against 50% found a value of *X*^2^ (1, *N* = 214) = 2.90, *p* = .004, meaning that performance was reliably *above* chance.

Confirmation Bias

In Study 1c, 130 of 214 participants (60.7%) thought the confirmation bias intervention would be effective. Given that Study 2b found no evidence that the confirmation bias intervention impacted performance, a one-proportion *Z* test against 50% found a value of *X*^2^ (1, *N* = 214) = 3.10, *p* = .002, meaning that performance was reliably *below* chance.

Delay

In Study 1c, 77 of 214 participants (36.0%) thought the response delay intervention would be effective. Given that Study 2b found evidence that the delay intervention impacted performance, a one-proportion *Z* test against 50% found a value of *X*^2^ (1, *N* = 214) = 4.10, *p* < .001, meaning that performance was reliably *below* chance.

Accuracy Reward

In Study 1c, 49 of 214 participants (22.9%) thought the accuracy reward intervention would be effective. Given that Study 2b found evidence that the accuracy intervention impacted performance, a one-proportion *Z* test against 50% found a value of *X*^2^ (1, *N* = 214) = 7.90, *p* < .001, meaning that performance was reliably *above* chance.

**Profile Correlations**

Ranking intervention effectiveness only on the basis of Study 2b results created a slightly different order than that found in the main text: 1) Delay, 2) Implementation Intentions, 3) Objectivity Commitment, 4) Confirmation Bias, 5) Accuracy Reward, 6) Accountability. In this case, the average profile correlation value was *q* = .104 (median: *q* = .143, 1^st^ quartile: *q* = -.257, 3^rd^ Quartile: *q* = .486).
